# Supplementary material for: Linear feedback coding scheme for multiple-access fading channels with degraded message sets
Source: PLoS One. 2024 Mar 18;19(3):e0295369. doi: 10.1371/journal.pone.0295369 (PMC10947716; doi:10.1371/journal.pone.0295369)
Supplement: S1 Appendix — (PDF) [file pone.0295369.s001.pdf]

A Proof of Lemma 1

For $2 \leq n \leq N$, define

$$\eta_{R,n}{}' = V_{R,n} + \frac{\eta_{R,n}}{b}. \tag{A1}$$

Note that

$$Var[\eta_{R,n}{}'^2] = E[(V_{R,n} + \frac{\eta_{R,n}}{b})^2] \overset{(a)}{=} E[V_{R,n}{}^2] + E[(\frac{\eta_{R,n}}{b})^2] \overset{(b)}{=} \frac{\sigma_1^2}{b^2} + (1-\rho^2)P_w, \tag{A2}$$

where (a) follows from the fact that $V_{R,n}$ is independent of $\eta_{R,n}$, (b) follows from (30). Furthermore, from (32) and (30), $V_{R,n}$ can be re-written as

$$\begin{aligned}
V_{R,n} &= \sqrt{\frac{(1-\rho^2)P_w}{\alpha_{n-1}{}'}}\varepsilon_{n-1}{}' V_{R,1} = \sqrt{\frac{(1-\rho^2)P_w}{\alpha_{n-1}{}'}\left(\varepsilon_{n-2}{}' - \frac{E[\frac{Y_{R,n-1}{}'}{b}\varepsilon_{n-2}{}']}{E[(\frac{Y_{R,n-1}{}'}{b})^2]}\frac{Y_{R,n-1}{}'}{b}\right)} \\
&\overset{(c)}{=} \sqrt{\frac{(1-\rho^2)P_w}{\alpha_{n-1}{}'}}\left(\varepsilon_{n-2}{}' - \frac{\sqrt{(1-\rho^2)P_w\alpha_{n-2}{}'}}{(1-\rho^2)P_w + \frac{\sigma_1^2}{b}}(V_{R,n-1} + \frac{\eta_{R,n-1}}{b})\right) \\
&\overset{(d)}{=} \sqrt{\frac{\alpha_{n-2}{}'}{\alpha_{n-1}{}'}}V_{R,k-1} - \sqrt{\frac{(1-\rho^2)P_w}{\alpha_{n-1}{}'}}\frac{\sqrt{(1-\rho^2)P_w\alpha_{n-2}{}'}}{(1-\rho^2)P_w + \frac{\sigma_1^2}{b}}(V_{R,n-1} + \frac{\eta_{R,k-1}}{b}) \\
&= \sqrt{\frac{\alpha_{n-2}{}'}{\alpha_{n-1}{}'}}V_{R,n-1} - \frac{(1-\rho^2)P_w}{(1-\rho^2)P_w + \frac{\sigma_1^2}{b^2}}\sqrt{\frac{\alpha_{n-2}{}'}{\alpha_{n-1}{}'}}(V_{R,n-1} + \frac{\eta_{R,n-1}}{b}) \\
&= \sqrt{\frac{\alpha_{n-2}{}'}{\alpha_{n-1}{}'}}V_{R,n-1} - \frac{b^2(1-\rho^2)P_w}{b^2(1-\rho^2)P_w + \sigma_1^2}\cdot\sqrt{\frac{\alpha_{n-2}{}'}{\alpha_{n-1}{}'}}V_{R,n-1} \\
&\quad - \frac{b^2(1-\rho^2)P_w}{b^2(1-\rho^2)P_w + \sigma_1^2}\cdot\sqrt{\frac{\alpha_{n-2}{}'}{\alpha_{n-1}{}'}}\frac{\eta_{R,n-1}}{b}
\end{aligned}$$

$$\overset{(e)}{=}\sqrt{\frac{\alpha_{n-2}{}'}{\alpha_{n-1}{}'}}\frac{\sigma_1^2}{b^2(1-\rho^2)P_w+\sigma_1^2}V_{R,n-1}-\sqrt{\frac{\alpha_{n-2}{}'}{\alpha_{n-1}{}'}}\frac{b^2(1-\rho^2)P_2}{b^2(1-\rho^2)P_2+\sigma_1^2}\frac{\eta_{R,n-1}}{b}, \tag{A3}$$

where (c) follows from $\varepsilon_{n-2}{}'$ is independent of $\eta_{R,n-1}$, $V_{R,n-1}=\sqrt{\frac{(1-\rho^2)P_w}{\alpha_{n-2}{}'}}\varepsilon_{n-2}{}'$ and $\alpha_{n-2}{}'\triangleq Var(\varepsilon_{n-2}{}')$, and (d) follows from $V_{R,n-1}=\sqrt{\frac{(1-\rho^2)P_w}{\alpha_{n-2}{}'}}\varepsilon_{n-2}{}'$, (e) follows from classical SK scheme [6]

$$\frac{\alpha_{n-1}{}'}{\alpha_n{}'}=\frac{b^2(1-\rho^2)P_w+\sigma_1^2}{\sigma_1^2}. \tag{A4}$$

Substituting (A3) into (A1), we have

$$\begin{aligned}\eta_{R,n}{}'&=\frac{\eta_{R,n}}{b}+V_{R,n}=\frac{\eta_{R,n}}{b}+\sqrt{\frac{\alpha_{n-2}{}'}{\alpha_{n-1}{}'}}\frac{\sigma_1^2}{b^2(1-\rho^2)P_w+\sigma_1^2}(V_{n-1}+\frac{\eta_{R,n-1}}{b})-\\&\sqrt{\frac{\alpha_{n-2}{}'}{\alpha_{n-1}{}'}}\cdot\frac{\eta_{R,n-1}}{b}\\&=\frac{\eta_{R,n}}{b}+\sqrt{\frac{\alpha_{n-2}{}'}{\alpha_{n-1}{}'}}\frac{\sigma_1^2}{b^2(1-\rho^2)P_w+\sigma_1^2}\eta_{R,n-1}{}'-\sqrt{\frac{\alpha_{n-2}{}'}{\alpha_{n-1}{}'}}\cdot\frac{\eta_{R,n-1}}{b}\\&=\frac{\eta_{R,n}}{b}+\frac{\sigma_1^2}{\sqrt{b^2(1-\rho^2)P_w+\sigma_1^2}}\eta_{R,n-1}{}'-\sqrt{\frac{b^2(1-\rho^2)P_w+\sigma_1^2}{\sigma_1^2}}\cdot\frac{\eta_{R,n-1}}{b}\end{aligned} \tag{A5}$$

At time 2, we have

$$\eta_{R,2}{}'=\frac{\eta_{R,2}}{b}+V_2=\frac{\eta_{R,2}}{b}+\eta_{R,1}\sqrt{\frac{(1-\rho^2)P_w}{b\sigma_1^2}}, \tag{A6}$$

V_2 is given by

$$\begin{aligned}V_2&=\sqrt{\frac{(1-\rho^2)P_w}{\alpha_1{}'}}\varepsilon_1{}'=\sqrt{(1-\rho^2)P_w}\cdot\sqrt{\frac{12b(1-\rho^2)P_w}{\sigma_1^2}}\cdot\frac{\eta_{R,1}}{b\sqrt{12(1-\rho^2)P_w}}\\&=\sqrt{\frac{(1-\rho^2)P_w}{b\sigma_1^2}}\eta_{R,1}.\end{aligned} \tag{A7}$$

On the other hand, we have

$$\begin{aligned}&E[Y_{R,n-1}\varepsilon_{n-2}]=E[(X_{n-1}^*+\eta_{R,n-1}{}')\varepsilon_{n-2}]\overset{(f)}{=}E[(\sqrt{\frac{P^*}{\alpha_{n-2}}}\varepsilon_{n-2}+\eta_{R,n-1}{}')\varepsilon_{n-2}]\\&=\sqrt{P^*\alpha_{n-2}}+E[\eta_{R,n-1}{}'\varepsilon_{n-2}],\end{aligned} \tag{A8}$$

(f) follows from $X_n^*=\frac{b}{a}U_{R,n}+X_{aR,n}=\sqrt{\frac{P^*}{\alpha_{n-1}}}\varepsilon_{n-1}$, and

$$\begin{aligned}E[(\frac{Y_{R,n-1}}{b})^2]&=E[(X_{n-1}^*+\eta_{R,n-1}{}')^2]\\&\overset{(g)}{=}P^*+2\sqrt{\frac{P^*}{\alpha_{n-2}}}E[\varepsilon_{n-2}\eta_{R,n-1}{}']+(1-\rho^2)P_w+\frac{\sigma_1^2}{b^2},\end{aligned} \tag{A9}$$

(g) follows from (A2). Based on (A8) and (A9), we have

$$\begin{aligned}\varepsilon_{n-1}&=\varepsilon_{n-2}-\frac{E[\frac{Y_{R,n-1}}{b}\varepsilon_{n-2}]}{E[(\frac{Y_{R,n-1}}{b})^2]}\frac{Y_{R,n-1}}{b}\\&=\varepsilon_{n-2}-\frac{\sqrt{P^*\alpha_{n-2}}+E[\varepsilon_{n-2}\eta_{R,n-1}{}']}{P^*+2\sqrt{\frac{P^*}{\alpha_{n-2}}}E[\varepsilon_{n-2}\eta_{R,n-1}{}']+(1-\rho^2)P_w+\frac{\sigma_1^2}{b^2}}\cdot\end{aligned}$$

$$
\begin{aligned}
&\left(\sqrt{\frac{P^*}{\alpha_{n-2}}}\varepsilon_{n-2}+{\eta_{R,n-1}}'\right)\\
&=\varepsilon_{n-2}\left(1-\frac{P^*+\sqrt{\frac{P^*}{\alpha_{n-2}}}E[\varepsilon_{n-2}{\eta_{R,n-1}}']}{P^*+2\sqrt{\frac{P^*}{\alpha_{n-2}}}E[\varepsilon_{n-2}{\eta_{R,n-1}}']+(1-\rho^2)P_w+\frac{\sigma_1^2}{b^2}}\right)\\
&-{\eta_{R,n-1}}'\cdot\frac{\sqrt{P^*\alpha_{n-2}}+E[\varepsilon_{n-2}{\eta_{R,n-1}}']}{P^*+2\sqrt{\frac{P^*}{\alpha_{n-2}}}E[\varepsilon_{n-2}{\eta_{R,n-1}}']+(1-\rho^2)P_w+\frac{\sigma_1^2}{b^2}}\\
&=\varepsilon_{n-2}\cdot\frac{\sqrt{\frac{P^*}{\alpha_{n-2}}}E[\varepsilon_{n-2}{\eta_{R,n-1}}']+(1-\rho^2)P_w+\frac{\sigma_1^2}{b^2}}{P^*+2\sqrt{\frac{P^*}{\alpha_{n-2}}}E[\varepsilon_{n-2}{\eta_{R,n-1}}']+(1-\rho^2)P_w+\frac{\sigma_1^2}{b^2}}\\
&-{\eta_{R,n-1}}'\cdot\frac{\sqrt{P^*\alpha_{n-2}}+E[\varepsilon_{n-2}{\eta_{R,n-1}}']}{P^*+2\sqrt{\frac{P^*}{\alpha_{n-2}}}E[\varepsilon_{n-2}{\eta_{R,n-1}}']+(1-\rho^2)P_w+\frac{\sigma_1^2}{b^2}}.
\end{aligned}
\tag{A10}
$$

Now from (A6) and (A10), we have

$$
\begin{aligned}
&E[\varepsilon_{n-1}{\eta_{R,n}}']\\
&=E[(\varepsilon_{n-2}\cdot\frac{\sqrt{\frac{P^*}{\alpha_{n-2}}}E[\varepsilon_{n-2}{\eta_{R,n-1}}']+(1-\rho^2)P_w+\frac{\sigma_1^2}{b^2}}{P^*+2\sqrt{\frac{P^*}{\alpha_{n-2}}}E[\varepsilon_{n-2}{\eta_{R,n-1}}']+(1-\rho^2)P_w+\frac{\sigma_1^2}{b^2}}\\
&-{\eta_{R,n-1}}'\cdot\frac{\sqrt{P^*\alpha_{n-2}}+E[\varepsilon_{n-2}{\eta_{R,n-1}}']}{P^*+2\sqrt{\frac{P^*}{\alpha_{n-2}}}E[\varepsilon_{n-2}{\eta_{R,n-1}}']+(1-\rho^2)P_w+\frac{\sigma_1^2}{b^2}})\cdot\\
&(\frac{\eta_{R,n}}{b}+\frac{\sigma_1^2}{\sqrt{b^2(1-\rho^2)P_w+\sigma_1^2}}{\eta_{R,n-1}}'-\sqrt{\frac{b^2(1-\rho^2)P_w+\sigma_1^2}{\sigma_1^2}}\cdot\frac{\eta_{R,n-1}}{b}]\\
&\overset{(h)}{=}E[\varepsilon_{n-2}{\eta_{R,n-1}}']\cdot\frac{\sigma_1^2}{\sqrt{b^2(1-\rho^2)P_w+\sigma_1^2}}\cdot\\
&\frac{\sqrt{\frac{P^*}{\alpha_{n-2}}}E[\varepsilon_{n-2}{\eta_{R,n-1}}']+(1-\rho^2)P_w+\frac{\sigma_1^2}{b^2}}{P^*+2\sqrt{\frac{P^*}{\alpha_{n-2}}}E[\varepsilon_{n-2}{\eta_{R,n-1}}']+(1-\rho^2)P_w+\frac{\sigma_1^2}{b^2}}\\
&-\frac{\sqrt{\frac{P^*}{\alpha_{n-2}}}E[\varepsilon_{n-2}{\eta_{R,n-1}}']+(1-\rho^2)P_w+\frac{\sigma_1^2}{b^2}}{P^*+2\sqrt{\frac{P^*}{\alpha_{n-2}}}E[\varepsilon_{n-2}{\eta_{R,n-1}}']+(1-\rho^2)P_w+\frac{\sigma_1^2}{b^2}}\frac{\sigma_1^2}{\sqrt{b^2(1-\rho^2)P_w+\sigma_1^2}}E[{\eta_{R,n-1}}'^2]\\
&+\frac{\sqrt{P^*\alpha_{n-2}}+E[\varepsilon_{n-2}{\eta_{R,n-1}}']}{P^*+2\sqrt{\frac{P^*}{\alpha_{n-2}}}E[\varepsilon_{n-2}{\eta_{R,n-1}}']+(1-\rho^2)P_w+\frac{\sigma_1^2}{b^2}}\cdot\\
&\sqrt{\frac{b^2(1-\rho^2)P_w+\sigma_1^2}{\sigma_1^2}}\cdot\frac{E[\eta_{R,n-1}{\eta_{R,n-1}}']}{b}\\
&\overset{(i)}{=}E[\varepsilon_{n-2}{\eta_{R,n-1}}']\cdot\frac{\sigma_1^2}{\sqrt{b^2(1-\rho^2)P_w+\sigma_1^2}}\cdot\\
&\frac{\sqrt{\frac{P^*}{\alpha_{n-2}}}E[\varepsilon_{n-2}{\eta_{R,n-1}}']+(1-\rho^2)P_w+\frac{\sigma_1^2}{b^2}}{P^*+2\sqrt{\frac{P^*}{\alpha_{n-2}}}E[\varepsilon_{n-2}{\eta_{R,n-1}}']+(1-\rho^2)P_w+\frac{\sigma_1^2}{b^2}},
\end{aligned}
\tag{A11}
$$

(h) follows from $E[\varepsilon_{n-2}\eta_{R,n-1}]=0$, and (i) follows from

$$
\frac{\sigma_1^2}{\sqrt{b^2(1-\rho^2)P_w+\sigma_1^2}}E[{\eta_{R,n-1}}'^2]=\sqrt{\frac{b^2(1-\rho^2)P_w+\sigma_1^2}{\sigma_1^2}}\cdot\frac{E[\eta_{R,n-1}{\eta_{R,n-1}}']}{b},
\tag{A12}
$$

where $E[{\eta_{R,n-1}'}^2]=\frac{\sigma_1^2}{b^2}+(1-\rho^2)P_w$, and $E[\eta_{R,n-1}\eta_{R,n-1}']=E[\eta_{R,n-1}(\frac{\eta_{R,n-1}}{b}+V_{R,n})]=\frac{\sigma_1^2}{b}$.

The first item of $E[\varepsilon_{n-1}\eta_{R,n}']$ is $E[\varepsilon_2\eta_{R,3}']$, and it is given by

$$\begin{aligned}
E[\varepsilon_2\eta_{R,3}'] &= E[\varepsilon_2(V_{R,3}+\frac{\eta_{R,3}}{b})] \\
&= E\left[\varepsilon_2(\frac{\eta_{R,3}}{b}+\sqrt{\frac{(1-\rho^2)P_w}{\alpha_2'}}\varepsilon_2')\right] \\
&\overset{(j)}{=} E[\frac{\sqrt{\frac{(1-\rho^2)P_w}{\sigma_1^2}}\eta_{R,1}+\frac{\eta_{R,2}}{b}}{\sqrt{12P^*}}\cdot(\frac{\eta_{R,3}}{b}+\sqrt{\frac{(1-\rho^2)P_w}{\alpha_2'}} \\
&(\frac{\sigma_1^2\cdot\eta_{R,1}}{12P_w(1-\rho^2)r^2}-\frac{\sigma_1\cdot\eta_{R,2}}{\sqrt{12}r^2}))] \\
&\overset{(k)}{=} \sqrt{\frac{(1-\rho^2)P_w}{12P^*\sigma_1^2}}E[{\eta_{R,1}}^2]\cdot\frac{\sigma_1^2}{12P_w(1-\rho^2)r^2}\sqrt{\frac{(1-\rho^2)P_w}{\alpha_2'}} \\
&-\sqrt{\frac{(1-\rho^2)P_w}{\alpha_2'}}\cdot\frac{E[{\eta_{R,2}}^2]}{b\sqrt{12P^*}}\cdot\frac{\sigma_1}{\sqrt{12}r^2}=0,
\end{aligned} \tag{A13}$$

where

$$r=\sqrt{b^2(1-\rho^2)P_w+\sigma_1^2}, \tag{A14}$$

(j) follows from $\alpha_2=\frac{b^2(1-\rho^2)P_w+\sigma_1^2}{12b^2P^*}$, and

$$\begin{aligned}
\varepsilon_2 &= \frac{V_{R,2}+\frac{\eta_{R,2}}{b}}{\sqrt{12P^*}}=\frac{\sqrt{\frac{(1-\rho^2)P_w}{\alpha_1'}}\varepsilon_1'+\frac{\eta_{R,2}}{b}}{\sqrt{12P^*}}=\frac{\sqrt{\frac{(1-\rho^2)P_w}{\sigma_1^2}}\eta_{R,1}+\frac{\eta_{R,2}}{b}}{\sqrt{12P^*}} \\
\varepsilon_2' &= \varepsilon_1'-\frac{E[\frac{Y_{R,2}'}{b}\varepsilon_1']}{E[(\frac{Y_{R,2}'}{b})^2]}\frac{Y_{R,2}'}{b} \\
&= \varepsilon_1'-\frac{E[(Y_{R,2}+\frac{\eta_{R,2}}{b})\varepsilon_1']}{(1-\rho^2)P_w+\frac{\sigma_1^2}{b}}(Y_{R,2}+\frac{\eta_{R,2}}{b}) \\
&= \varepsilon_1'-\frac{b^2E[(\sqrt{\frac{(1-\rho^2)P_w}{\alpha_1'}}\varepsilon_1'+\frac{\eta_{R,2}}{b})\varepsilon_1']}{r^2} \\
&(\sqrt{\frac{(1-\rho^2)P_w}{\alpha_1'}}\varepsilon_1'+\frac{\eta_{R,2}}{b}) \\
&= \varepsilon_1'-\frac{b^2\sqrt{(1-\rho^2)P_w\alpha_1'}+\frac{\eta_{R,2}}{b}}{r^2}(\sqrt{\frac{(1-\rho^2)P_w}{\alpha_1'}}\varepsilon_1'+\frac{\eta_{R,2}}{b}) \\
&= \varepsilon_1'(1-\frac{b^2(1-\rho^2)P_w}{r^2})-\frac{b\sqrt{(1-\rho^2)P_w\alpha_1'}\cdot\eta_{R,2}}{r^2} \\
&= \frac{\sigma_1^2}{br^2\sqrt{12(1-\rho^2)P_w}}\eta_{R,1}-\frac{\sigma_1}{r^2\sqrt{12}}\eta_{R,2}.
\end{aligned} \tag{A15}$$

Now substituting (A13) into (A11), we can conclude that $E[\varepsilon_{n-1}\eta_{R,n}']=0$ for all $3\le n\le N$. Then we have

$$\begin{aligned}
\varepsilon_n &= \varepsilon_{n-1}-\frac{E[\frac{Y_{R,n}}{b}\varepsilon_{n-1}]}{E[(\frac{Y_{R,n}}{b})^2]}\frac{Y_{R,n}}{b} \\
&= \varepsilon_{n-1}-\frac{\sqrt{P^*\alpha_{n-1}}+E[\varepsilon_{n-1}\eta_{R,n}']}{P^*+2\sqrt{\frac{P^*}{\alpha_{n-1}}}E[\varepsilon_{n-1}\eta_{R,n}']+(1-\rho^2)P_w+\frac{\sigma_1^2}{b^2}}\cdot \\
&\left(\sqrt{\frac{P^*}{\alpha_{n-1}}}\varepsilon_{n-1}+\eta_{R,n}'\right)
\end{aligned}$$

$$= \varepsilon_{n-1} \left(1 - \frac{P^* + \sqrt{\frac{P^*}{\alpha_{n-1}}} E[\varepsilon_{n-1} \eta_{R,n}']}{P^* + 2\sqrt{\frac{P^*}{\alpha_{n-1}}} E[\varepsilon_{n-1}\eta_{R,n}'] + (1-\rho^2)P_w + \frac{\sigma_1^2}{b^2}} \right)$$

$$-\eta_{R,n}' \cdot \frac{\sqrt{P^* \alpha_{n-1}} + E[\varepsilon_{n-1}\eta_{R,n}']}{P^* + 2\sqrt{\frac{P^*}{\alpha_{n-1}}} E[\varepsilon_{n-1}\eta_{R,n}'] + (1-\rho^2)P_w + \frac{\sigma_1^2}{b^2}}$$

$$= \varepsilon_{n-1} \cdot \frac{\sqrt{\frac{P^*}{\alpha_{n-1}}} E[\varepsilon_{n-1}\eta_{R,n}'] + (1-\rho^2)P_w + \frac{\sigma_1^2}{b^2}}{P^* + 2\sqrt{\frac{P^*}{\alpha_{n-1}}} E[\varepsilon_{n-1}\eta_{R,n}'] + (1-\rho^2)P_w + \frac{\sigma_1^2}{b^2}}$$

$$-\eta_{R,n-1}' \cdot \frac{\sqrt{P^* \alpha_{n-1}} + E[\varepsilon_{n-1}\eta_{R,n}']}{P^* + 2\sqrt{\frac{P^*}{\alpha_{n-1}}} E[\varepsilon_{n-1}\eta_{R,n}'] + (1-\rho^2)P_w + \frac{\sigma_1^2}{b^2}}$$

$$= \varepsilon_{n-1} \cdot \frac{(1-\rho^2)P_w + \frac{\sigma_1^2}{b^2}}{P^* + (1-\rho^2)P_w + \frac{\sigma_1^2}{b^2}} - \eta_{R,n}' \frac{\sqrt{P^* \alpha_{n-1}}}{P^* + (1-\rho^2)P_w + \frac{\sigma_1^2}{b^2}}$$

$$= \varepsilon_{n-1} \cdot \frac{r^2}{b^2 P^* + r^2} - \eta_{R,n}' \cdot \frac{b^2 \sqrt{P^* \alpha_{n-1}}}{b^2 P^* + r^2}, \tag{A16}$$

substituting (A16) into $\alpha_{n-1} \triangleq Var(\varepsilon_{n-1})$, we have

$$\alpha_n = Var(\varepsilon_n) = Var(\varepsilon_{n-1} \cdot \frac{r^2}{b^2 P^* + r^2} - \eta_{R,n}' \cdot \frac{b^2 \sqrt{P^* \alpha_{n-1}}}{b^2 P^* + r^2})$$

$$= E[{\varepsilon_n}'^2] \frac{r^4}{(b^2 P^* + r^2)^2} - 2E[\varepsilon_{n-1}\eta_{R,n}'] \frac{r^2}{b^2 P^* + r^2} \frac{b^2 \sqrt{P^* \alpha_{n-1}}}{b^2 P^* + r^2} +$$

$$E[\eta_{R,n}'^2] \frac{b^4 P^* \alpha_{n-1}}{(b^2 P^* + r^2)^2} = \alpha_{n-1} \frac{r^4}{(b^2 P^* + r^2)^2} + \frac{r^2}{b^2} \cdot \frac{b^4 P^* \alpha_{n-1}}{(b^2 P^* + r^2)^2}$$

$$= \alpha_{n-1} \cdot \frac{r^2}{b^2 P^* + r^2}. \tag{A17}$$

Based on the classical SK [6], we have

$$P_{e,aR} \leq 2Q[\frac{1}{2 \cdot 2^{NR_{aR}(N,\varepsilon)}} \cdot (\frac{r^2}{b^2 P^* + r^2})^{-\frac{N-1}{2}} \cdot {\alpha_2}^{-\frac{1}{2}}]$$

$$\Rightarrow P_{e,aR} \leq 2Q[\frac{b\sqrt{12P^*}}{2\sqrt{b^2 P^* + r}} \cdot 2^{N(\frac{1}{2}\log(1+\frac{b^2 P^* + r^2}{r^2}) - R_{aR}(N,\varepsilon))}]. \tag{A18}$$

Let $P_{e,aR} = P_{e,aI} = \frac{\varepsilon}{4}$ (here $P_{e,aR}$ and $P_{e,aI}$ are the decoding error probabilities of the sub-messages W_{aR} and W_{aI}, respectively), i.e., $P_{e,a} = \frac{\varepsilon}{2}$, therefore, for given decoding error probability ε and codeword length N, we have

$$R_{aR}(N,\varepsilon) = \frac{1}{2}\log(1 + \frac{b^2 P^*}{r^2}) - \frac{1}{2N}\log(\frac{b^2 P^* + r^2}{3b^2 P^*} \cdot [Q^{-1}(\frac{\varepsilon}{8})]^2). \tag{A19}$$

Analogously, we conclude that

$$R_{bR}(N,\varepsilon) = \frac{1}{2}\log(1 + \frac{b^2(1-\rho^2)P_w}{\sigma_1^2}) - \frac{1}{2N}\log(\frac{b^2(1-\rho^2)P_w + \sigma_1^2}{3b^2(1-\rho^2)P_w} \cdot [Q^{-1}(\frac{\varepsilon}{8})]^2). \tag{A20}$$

By symmetry, we have $R_{aR}(N,\varepsilon) = R_{aI}(N,\varepsilon)$ and $R_{bR}(N,\varepsilon) = R_{bI}(N,\varepsilon)$, finally, the transmission sum rate $R_{sum}(N,\varepsilon) = 2R_{aR}(N,\varepsilon) + 2R_{bR}(N,\varepsilon)$ is obtained, which completes the proof.
