# Supplementary material for: Linear feedback coding scheme for multiple-access fading channels with degraded message sets
Source: PLoS One. 2024 Mar 18;19(3):e0295369. doi: 10.1371/journal.pone.0295369 (PMC10947716; doi:10.1371/journal.pone.0295369)
Supplement: Attachment — Submitted filename: response-plos one.pdf [file pone.0295369.s002.pdf]

Response to Reviewers Report on PONE-D-23-28003: *Linear Feedback Coding Scheme for Multiple-access Fading Channels with Degraded Message Sets*

Yuan Liao, and Xiaofang Wang

We would like to thank the Editor, Prof. Praveen Kumar Donta, for allowing a resubmission of our manuscript, with an opportunity to address the reviewers' comments. We have carefully revised the manuscript according to these comments, which we sincerely appreciate as they help us to improve the quality of the manuscript. A point-by-point response to the comments of the reviewers is attached below.

CONTENTS

I **Reply to Reviewer 1** 2

II **Reply to Reviewer 2** 4

III **Reply to Reviewer 3** 5

References 6

I. Reply to Reviewer 1

Q1) "In Lines 52-56, the introduction of sections is missing."

Answer: Thank you very much for the comment! According to the reviewer's suggestion, we add the introduction of Sections in Lines 50-57, see the highlights of the revised manuscript.

Q2)"Why there are two repeated SK feedback scheme for the FMAC-DMS in Line 108 and 109?"

Answer: Thank you very much for the comment! In revised manuscript, this kind of problems are corrected.

Q3) "In Line 277 and Line 278, please be careful about English writing From this figure, According to the..."

Answer: Thank you very much for the comment! We have checked through the manuscript and corrected this kind of problems as suggested, see the highlights of the revised manuscript.

Q4) "In figure, you use Fig. But in description, you use Figure. Please unify your expression."

Answer: Thank you very much for the comment! According to the reviewer's suggestion, we have standardized the expression of "Fig." and "Figure" to "Fig.".

Q5) "From Figure 6, we see that the required coding blocklength achieving desired decoding error probability is significantly shorter than LDPC, Turbo and LDGM codes. But I find that after 39 SNR(dB), the LDPC code has shorter error probability than your proposed model. It seems that the trend of plots is different from your conclusion. Could you give some explanations?"

Answer: Thank you very much for the comment! According to the reviewer's comment, we have thoroughly reviewed our simulation program. We corrected parameter errors in our program and provided new simulation results, as shown in Fig. 1. From Fig. 1, we see that the coding blocklength required to achieve the desired decoding error probability is significantly shorter than other coding schemes.

Q6) "What are the advantages of your channel coding scheme? What are the contributions of your coding scheme in high reliability, low latency and data transmission security of FMAC-DMS? Please add more experiments to support your conclusions."

Answer: Thank you very much for the comment! We have added and modified Fig. 2, Fig. 3 and Fig. 4 in this revised manuscript. From Fig. 2, we see that the required coding blocklength for achieving the desired decoding error probability is significantly shorter than the LDPC, Turbo, and LDGM codes, which indicates that our proposed linear feedback coding scheme is a high reliability and low latency coding scheme (the shorter coding blocklength, the lower latency in communication). From Fig. 3, we conclude that the lower decoding error probability and higher secrecy level lead to larger blocklength threshold satisfying secrecy constraint. For example, when the decoding error probability $\epsilon = 10^{-7}$

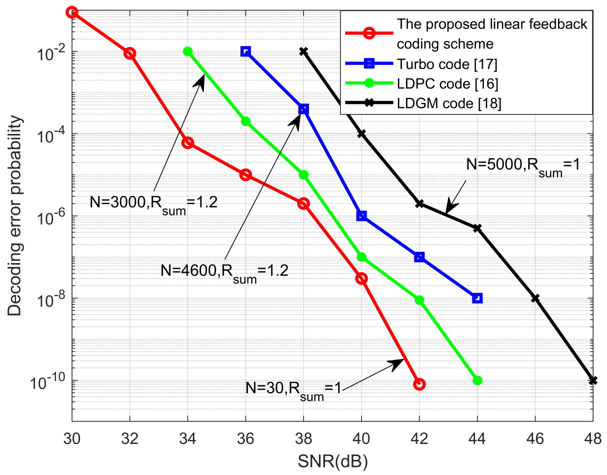

Fig. 1: Comparison of the SNR and decoding error probability of the LDPC code, Turbo code, LDGM code and the proposed linear feedback coding scheme for the FMAC-DMS.

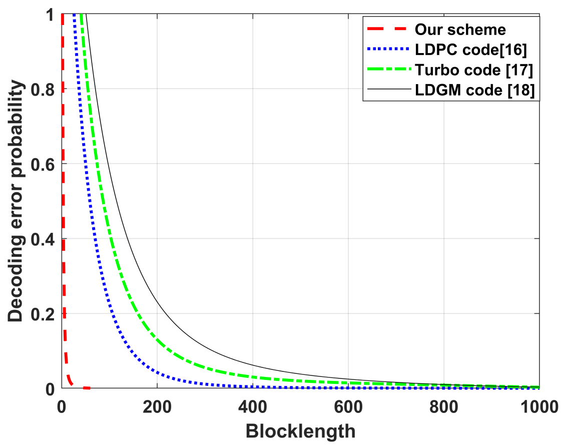

Fig. 2: Comparison of the blocklength and decoding error probability of the LDPC code, Turbo code, LDGM code and the proposed linear feedback coding scheme for the FMAC-DMS.

and the secrecy level $\delta = 0.99$, the blocklength threshold satisfying secrecy constraint is about 400. In Fig. 4, we add the simulation result of LDPC code [1] based on the secret key scheme [2], [3] (message encrypted by the key). Fig. 4 shows that the sum rate of our scheme is higher than that of LDPC code when the decoding error probability is the same. Based on the aforementioned results, our proposed coding scheme almost achieves perfect secrecy when the blocklength is larger than the blocklength threshold satisfying secrecy constraint. Furthermore, our proposed coding scheme shows higher reliability and lower latency (shorter blocklength) compared to other schemes.

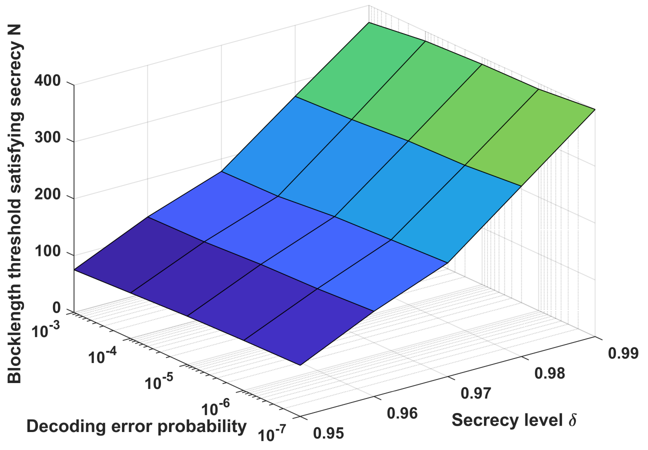

Fig. 3: The relationship between the blocklength threshold satisfying secrecy constraint, the decoding error probability, and the secrecy level for the MISO-FMAC-DMS and external eavesdropper.

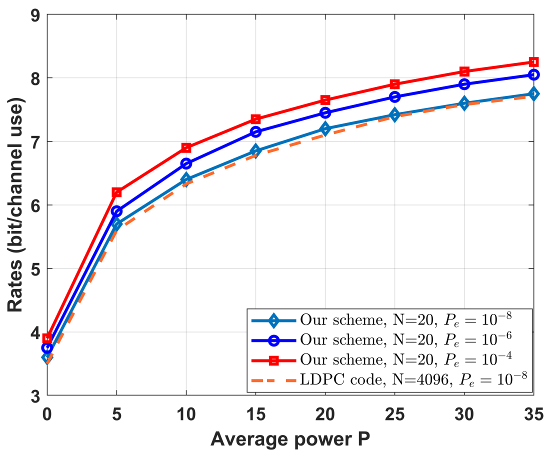

Fig. 4: The relationship between the sum rate, decoding error probability and power constraint for the MISO-FMAC-DMS.

II. Reply to Reviewer 2

Q1) "This presents the main idea of the layered SK scheme and applies it to FMAC-DMS in both single-input single-output (SISO) and multi-input single-output antenna systems (MISO). The channel capacities and FBL achievable rate expressions for both scenarios are derived. The FBL achievable secure rate expressions are also analyzed. All the objectives are well addressed in a systematic way with a proper English. Congratulations to the authors for the good work."

Answer: We appreciate the reviewer's positive comments on this paper. However, as pointed out by

other reviewers, the quality of this manuscript still needs to be significantly improved.

III. Reply to Reviewer 3

Q1) "Please avoid using first-person sentences. Use instead third or passive-voice sentences."

Answer: Thank you very much for the comment! We have carefully reviewed the manuscript and have replaced third-person or passive-voice sentences with first-person sentences, see the highlights of the revised manuscript.

Q2) "I suggest to improve the Abstract section. This section should give a pertinent overview of the work by presenting a brief background of the work, methods, results and conclusion. Also, present quantitative results in order to highlight the novelty of the work."

Answer: Thank you very much for the comment! In this revised manuscript, we revise the Abstract section according to the reviewer's suggestion, see the details below.

Channel coding plays an important role in wireless communication systems, and it serves as a crucial mechanism to reduce interference during the transmission process. As the fifth-generation (5G) and sixth-generation (6G) wireless communication systems rapidly advance, requirements of the users on the quality and security of wireless service are increasing. To solve these problems, exploring new channel coding technologies is necessary. In this paper, a linear feedback coding scheme for fading multiple-access channels with degraded message sets (FMAC-DMS) is proposed. In this scheme, the transmitting beamforming and channel splitting are used to transform the channel with complex signals into scalar equivalent sub-channels. Then, the extended Schalkwijk-Kailath coding scheme (SK) is further applied to each sub-channel. The channel capacity, finite blocklength (FBL) sum-rate and FBL secrecy achievable sum-rate of the FMAC-DMS in single-input single-output (SISO) and multi-input single-output (MISO) cases are derived. Finally, we show that the proposed scheme not only provides a FBL coding solution but also guarantees physical layer security(PLS). Numerical and simulation results show the effectiveness of the proposed scheme as a channel coding solution. The study of this paper provides a new method to construct a practical FBL scheme for the FMAC-DMS.

Q3) "Improve image quality."

Answer: Thank you very much for the comment! This kind of problems are corrected.

Q4) "Validate that all variables and parameters of the equations are correctly defined."

Answer: Thank you very much for the comment! In this revised manuscript, all variables and parameters in the equations have been correctly defined, see the highlights of the revised manuscript.

Q5) "The numbering of the sections that make up the writing needs to be displayed correctly."

Answer: Thank you very much for the comment! In this revised manuscript, the section numbering has been correctly displayed.

Q6) "Also, I suggest to add a Discussion section. Here, add a table that compares the proposed work vs the already reported in the state-of-the-art in order to highlight the contributions of the work."

Answer: Thank you very much for the comment! As suggested, we added a Discussion section and a table that compares our work to previous works to highlight the contribution of this paper, see Section II of this revised manuscript.

Q7) "Please update your references."

Answer: Thank you very much for the comment! In this revised manuscript, the references have been correctly updated, see References Section of this revised manuscript.

Q8) "Put a brief introduction between sections and subsections."

Answer: Thank you very much for the comment! We have appropriately added brief introductions between sections and subsections according to the reviewer's suggestion, see the highlights of the revised manuscript.

Finally, the authors would like to thank the editor and all the reviewers again for their valuable suggestions on improving this paper.

References

[1] Y. Li, L. Wang and Y. Sun, "The Design of Network Low Density Parity Check Codes for Wireless Multiple-Access Relay Networks," *2011 IEEE International Conference on Advanced Information Networking and Applications*, Biopolis, Singapore, 2011, pp. 653-658.

[2] Ahlswede. R and N. Cai, "Transmission, identification and common randomness capacities for wire-tap channels with secure feedback from the decoder," *Gen. Theory Inf. Trans. Comb.*, pp. 258-275, 2006.

[3] R. F. Schaefer, A. Khisti and H. V. Poor, "Secure Broadcasting Using Independent Secret Keys," *IEEE Transactions on Communications*, vol. 66, no. 2, pp. 644-661, Feb. 2018.
