# Supplementary material for: Linear feedback coding scheme for multiple-access fading channels with degraded message sets
Source: PLoS One. 2024 Mar 18;19(3):e0295369. doi: 10.1371/journal.pone.0295369 (PMC10947716; doi:10.1371/journal.pone.0295369)
Supplement: Attachment — Submitted filename: response letter-v2.pdf [file pone.0295369.s003.pdf]

Response to Reviewers Report on PONE-D-23-28003R1: *Linear Feedback Coding Scheme for Multiple-access Fading Channels with Degraded Message Sets*

Yuan Liao, and Xiaofang Wang

We would like to thank the Editor, Prof. Praveen Kumar Donta, for allowing a resubmission of our manuscript, with an opportunity to address the reviewers' comments. We have carefully revised the manuscript according to these comments, which we sincerely appreciate as they help us to improve the quality of the manuscript. A point-by-point response to the comments is attached below.

CONTENTS

I Reply to Editor Comments 2

I. Reply to Editor Comments

Q1) "Summarize all notations used in this paper through a table. Since many notations, summary table helps to cross check when needed."

Answer: Thank you very much for the comment! According to the editor's suggestion, we added the summary table of key notations in Section 3, see Section 3 of this revised manuscript.

Q2)"From line 163 (page 6/24) to rest of the document is in italic. please rectify it."

Answer: Thank you very much for the comment! In this revised manuscript, this kind of problems are corrected.

Q3) "Simulation parameters are summarized through a table. It is recommended to provide the source code of simulation through a link."

Answer: Thank you very much for the comment! According to the editor's suggestion, we added the summary table of simulation parameters in Section 6, see Section 6 of this revised manuscript. In addition, providing the source code link is not necessary. Our simulation results are based on Monte Carlo method using Matlab, and the source code for the proposed coding scheme can be straightforwardly implemented based on the coding procedure provided in the paper (see Fig. 1). Furthermore, the source code for other coding schemes is already well-established in Matlab.

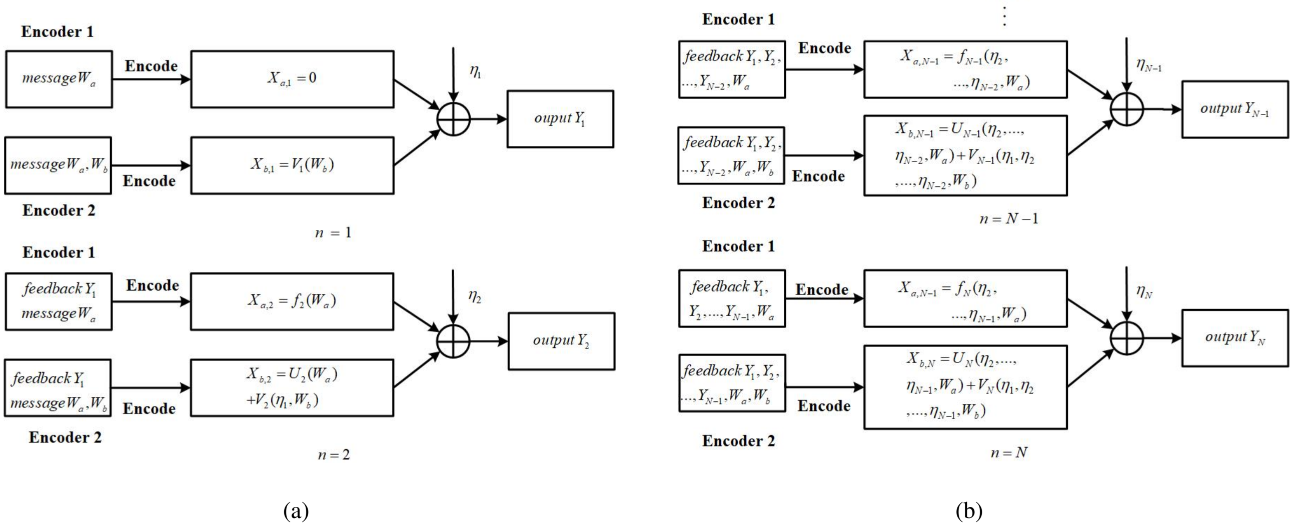

Fig. 1: Procedure of our proposed SK-type scheme.

Q4) "Discussions related to the plots is very limited. Recommended to provide sufficient discussion for each plot describing axis, reasons for improvements of proposed work over others, challenges etc."

Answer: Thank you very much for the comment! In this revised manuscript, we revise the discussions related to the plots according to the editor's suggestion, see the highlights of the revised manuscript.

Finally, the authors would like to thank the editor and all the reviewers again for their valuable suggestions on improving this paper.
